# Pre-clinical medical student reflections on implicit bias: Implications for learning and teaching

**DOI:** 10.1371/journal.pone.0225058

**Published:** 2019-11-15

**Authors:** Christine Motzkus, Racquel J. Wells, Xingyue Wang, Sonia Chimienti, Deborah Plummer, Janice Sabin, Jeroan Allison, Suzanne Cashman

**Affiliations:** 1 Clinical and Population Health Research, University of Massachusetts Medical School, Worcester, MA, United States of America; 2 Division of Nephrology, Duke University, Durham, NC, United States of America; 3 Department of Family Medicine, University of Washington Medical School, Seattle, WA, United States of America; 4 Office of Student Affairs, University of Massachusetts Medical School, Worcester, MA, United States of America; 5 Department of Psychiatry, University of Massachusetts Medical School, Worcester, MA, United States of America; 6 Department of Biomedical Informatics and Medical Education, University of Washington, School of Medicine, Seattle, WA, United States of America; 7 Department of Population and Quantitative Health Sciences University of Massachusetts Medical School, Worcester, MA, United States of America; 8 Department of Family Medicine and Community Health, University of Massachusetts Medical School, Worcester, MA, United States of America; East Carolina University Brody School of Medicine, UNITED STATES

## Abstract

**Context:**

Implicit bias affects health professionals’ clinical decision-making; nevertheless, published reports of medical education curricula exploring this concept have been limited. This research documents a recent approach to teaching implicit bias.

**Methods:**

Medical students matriculating during 2014 and 2015 participated in a determinants of health course including instruction about implicit bias. Each submitted a reflective essay discussing implicit bias, the experience of taking the Implicit Association Test (IAT), and other course content. Using grounded theory methodology, student essays that discussed reactions to the IAT were analyzed for content themes based on specific statements mapping to each theme. Twenty-five percent of essays underwent a second review to calculate agreement between raters regarding identification of statements mapping to themes.

**Outcome:**

Of 250 essays, three-quarters discussed students’ results on the IAT. Theme comments related to: a) experience taking the IAT, b) bias in medicine, and c) prescriptive comments. Most of the comments (84%) related to students’ acknowledging the importance of recognizing implicit bias. More than one-half (60%) noted that bias affects clinical decision-making, and one-fifth (19%) stated that they believe it is the physician’s responsibility to advocate for dismantling bias.

**Conclusions:**

Through taking the IAT and developing an understanding of implicit bias, medical students can gain insight into the effect it may have on clinical decision-making. Having pre-clinical medical students explore implicit bias through the IAT can lay a foundation for discussing this very human tendency.

## Introduction

Implicit bias, also known as hidden or unconscious bias, refers to the attitudes or stereotypes that affect our understanding, actions, and decisions in a non-conscious manner of which we are typically unaware [1–4]. Implicit biases are automatic, lower level beliefs that can be difficult to recognize, acknowledge and manage [1,5]. Implicit bias is distinct from explicit biases which are our attitudes and beliefs which we consciously endorse and can report as our beliefs. In the health professions, implicit bias has been documented among medical students [6] as well as among resident and attending physicians, who routinely assume the role of medical education instructors and who serve as role models to students [7,8]. Recognition of implicit bias has been shown to produce dissonance for health professionals as they consider their conscious beliefs versus their non-conscious selves [9]. Becoming aware of one’s own implicit bias results may result in a disconnect between the clinician’s idealized desire to treat all patients equitably and actual clinical decisions that are influenced by unconscious attitudes towards a patient based on his/her subgroup identity. It has been well documented that in clinical settings, negative implicit bias leads to poorer quality of care, poor or inadequate clinician-patient communication, and ultimately to healthcare disparities and inequities [8,10–14].

For more than a decade, medical education leaders have urged medical education to embrace cultural competence instruction [4], including training about how to manage unconscious bias, thus introducing learners to this foundational issue that affects quality of care. This instruction sets an expectation that students consider their biases when caring for patients [13]. For students to learn how to address implicit bias, however, they must first be open to confronting the fact that they likely have biases functioning at an unconscious level and to recognizing the potential effect these biases may have [4]. This can be particularly challenging when evidence suggests one’s own implicit bias conflicts with one’s personal or professional identity [9,14].

One approach used to self-identify unconscious biases is the Implicit Association Test (IAT) [15]. Increasingly, health professions schools are using the IAT to introduce the issue of bias and stereotype [16]. Results from a recent study using the IAT with a small group of physicians and nurses emphasized the necessity of understanding how individuals process and integrate their IAT results [17]. Studies have suggested that improving our understanding about how individuals react to their IAT results should increase our ability to use the IAT to manage bias by promoting self-awareness as well as contribute to personal and professional development [17–19].

While the IAT is being used more frequently in health professions education, the additional step of asking medical students to reflect in writing on the outcome of their self-assessment is less common, and reports on these reflections in the literature are rarer still. Given the power of reflective practice [20] and its use in medical education to raise awareness of bias [7,17,20–22], we hypothesized that we could gain insight into understanding students’ readiness to accept their implicit biases and to address unconscious bias from their written responses reflecting on their assessment results. Using grounded theory methodology, we examined the content of two cohorts of one school’s first year medical students’ required written reflections on their IAT results. We explored whether three-hours of focused content and discussion regarding bias and stereotypes could produce student reflections demonstrating insight into these issues. We were particularly curious about whether students would include comments indicating that 1) they believe bias has the potential to influence decisions within their newly chosen profession and 2) where responsibility lay for reducing bias’s effects.

## Methods

### Determinants of health course

All first and second year medical students at University of Massachusetts Medical School (UMMS) are enrolled in the course: Determinants of Health (DOH). This required course provides students with a systematic framework for understanding the disparities and inequities found in humans’ health status as well as the impact of context/environment on health. For first-year students, DOH consists of five hours of lecture/discussion in the fall semester and three in the spring, with an Epidemiology/Biostatistics module taking place in the winter.

Bias/stereotyping is a determinant of health that is featured as part of DOH’s three-hour spring session. Complementing their learning regarding how our brains receive and catalogue information that results in stereotyping, students are asked to complete the Implicit Association Test (IAT) [15] on any topic of their choosing (e.g. race, religion, weight). We used the IAT with feedback as a tool for self-reflection. Research in healthcare shows that the IAT may engender critique of its validity and defensiveness, yet it is considered to be either a positive or neutral experience and provokes test takers to acknowledge the need to address their biases [23]. The IAT is a computerized test that measures the strength of association between target concepts such as race (Black versus White), ethnicity (Hispanic/Latino versus White), or body type (X versus Y) with either pleasant or unpleasant words. Test takers are asked to rapidly sort and pair images and words as they appear on a computer screen using a right and left key. While the main idea of the IAT is that response time is faster when items are closely associated and slower when they are not, it should not be used as a diagnostic of prejudicial or discriminatory behavior.

### Reflective essays

One requirement for completing the DOH course’s spring segment is that each student compose and submit a two-page reflective essay in response to the prompt “*Please select a particular reading assigned for this class*, *your experience taking the Implicit Association Test*, *or a specific discussion that occurred in class and comment on how this material leads to new insight about the potential effect of your personal biases or stereotyping on future clinical decisions*.” To meet this study’s objectives, we de-identified each essay that students in the 2014–2015 and 2015–2016 academic years submitted, selected those commenting on the IAT (n = 188; 74%), and used qualitative analytic techniques for analysis.

### Qualitative analysis

Grounded theory methodology was used to anchor the qualitative analysis of students’ essays. With its inductive approach, grounded theory is a systematic research methodology that uses the analysis of data to construct theory. Studies using grounded theory begin with the collection of qualitative data. In reading and re-reading the data, analysts identify ideas or concepts that appear repeatedly. Text is tagged with codes which are noted in a code book and then grouped into emerging themes [24].

One author (RW) developed the codebook for the first cohort of essays through an iterative process of essay review until saturation of content was reached. The codebook was organized such that specific statements (typically sentences or clauses) were mapped to one of 25 themes identified. A student could make multiple statements in an essay that mapped to the same theme; however, themes were not double counted for this repetition. Themes were loosely grouped under three broader “thematic categories” including: Experience with the Implicit, Bias in Medicine, and Prescriptive Comments. The codebook was reviewed by a second author (CM) with discussion to ensure the scope of the codebook was adequate. The second cohort’s essays were reviewed using the initial codebook by one author (XW) undergoing the same process of mapping statements to themes.

### Inter-rater reliability

Twenty-five percent from each of the two years of essays reflecting on the IAT experience were randomly selected using a random number generator to undergo coding by a second rater (CM). These essays were specifically reviewed to identify statements that mapped to a theme that may have been missed by initial review or statements that the second rater felt had been misidentified as corresponding to a theme. Using Cohen’s Kappa statistic in SAS 9.3 (Cary,NC), inter-rater reliability was calculated for rater agreement.

### Ethics statement

As the University of Massachusetts Institutional Review Board deemed this research not human subjects research, the informed consent requirement was waived.

## Results

A total of 250 student essays were de-identified by DOH faculty leaders. Slightly more than one-half (135) of these two medical student cohorts were women; their average age was 24, and 35 were first generation to attend college. Twenty-six were from groups underrepresented in medicine. With the exception of 12 of the 18 MD/PhD students in this cohort, all were residents of Massachusetts.

Approximately three-quarters (74%) of the essays mapped directly to discussion of the IAT and the student’s IAT results. For the final code book, 25 themes were identified. (See Table 1) Themes related to the experience of taking the IAT included recognition of bias’s inevitability, as bias is a product of society, cultural backgrounds, and media. Themes related to the potential influence of implicit bias in medicine included comments on the existence of racial bias in medicine and the harm that can be caused by the inability to acknowledge bias. Finally, several themes supported the prescriptive ideas that in order to provide high quality patient care, bias must be recognized and that it is a physician’s responsibility to dismantle the bias found in the healthcare system through avenues such as advocacy and legislation. Inter-rater reliability was high (kappa = 0.87, p<0.0001).

**Table 1:** Percentage of student essay content mapping to three categories and their themes.

Category	Percentage of Students Identifying Theme (n = 250)
**Experience with the IAT**	
IAT promotes self-reflection.	56
Bias is a product of society/cultural background/media/upbringing.	55
Bias is inevitable/exists.	54
Validity of the IAT	29
IAT results were surprising to the student.	28
IAT is a beneficial tool to acknowledge racial bias.	27
Bias is not always acted upon.	14
IAT results were not surprising to the student.	13
**Bias in Medicine**	
Bias affects decision making with regard to patient care.	60
Racial bias/racism exists in medicine.	51
Current inequalities have impact on the medical care.	38
Systemic/Institutional bias is outside the bias of the individual physician.	31
Historic events have affected the climate of the medical field.	18
Inability to acknowledge bias or identifying as colorblind is harmful.	16
A fine line exists between clinical judgement and bias for patient care.	8
The majority and minority have similar associations/bias.	6
Passive reinforcement of implicit bias occurs in medical education.	6
**Prescriptive Comments**	
Bias needs to be acknowledged and recognized.	84
Addressing bias requires conscious effort and self-reflection.	74
Action is needed for focusing on the problems causing disparities and combating institutional bias.	22
Discussion on bias is needed.	21
Physicians should be advocates for policy and legislative impact.	18
Physicians should hold colleagues accountable.	7
Objectivity allows us to combat bias.	6
Physicians need concrete evidence and research to move forward with combating racism in medicine.	4

We identified 10 specific themes that reflected broadly on the general concept of *students’ experience with the IAT*. Promisingly, slightly more than one-half of students (56%) felt that the IAT promoted self-reflection. More than one-quarter (28%) wrote that their IAT results surprised them. The students who professed surprise generally noted one of two sentiments. Either surprise was the reaction to discovering biases that seemed to run counter to their own self-identification, or it was surprising that they harbored any form of prejudice.

“The most shocking part of taking the test, however, was how official it made my prejudices.”

More than one-half of students (55%) acknowledged the inevitability of personal biases and that these biases—whether acknowledged or unacknowledged—affect behavior. Most (56%) also acknowledged that bias is a product of society/cultural background/media/upbringing; these students discussed the importance of recognizing the impact of their own background and experiences.

“It's OK to admit that we are not perfect. It's OK to recognize the fear and vulnerability that comes with realizing our personal prejudices. Because we're all raised differently under different circumstances, and because biases are natural and undeniable.”

Overall, comments regarding bias and the IAT generally reflected acceptance of biases and the importance of self-awareness.

“Every person has certain impressions and biases that they are not proud of, but the lesson to take away from tests like the Implicit Association Test is to acknowledge these associations and separate them from their actions.”

Students frequently commented on areas of bias in clinical medicine and medical education that mapped to themes that related to the *influence of bias on health care*. While slightly more than one-half (52%) acknowledged that racial bias/racism exists in medicine, three-fifths (60%) stated that they felt bias affected patient care decision-making.

“We all have some degree of inherent bias whether we are aware of it or not and those subtle subconscious decisions we make on a daily basis can have a large impact on others.”

Almost one-fifth (18%) identified historic events as having affected the medical field’s overall climate related to bias and stereotype. A common thread throughout the essays was surprise at the pervasiveness of racial bias within systems students had previously believed to be objective. Some expressed dismay that historical events they considered quite distant are still frequently recalled by patients and that as a result, likely perpetuate inequities.

“Historical events institutionalized racism into American society, and remnants of prejudiced attitudes and differential treatment translate into gaps in health care outcomes across racial groups.”

“Prior to this class, I had never thought about how racial biases could be manifested within such a seemingly objective and technical system such as the organ procurement organization.”

A small proportion (8%) of students also highlighted the difficulty of balancing clinical judgement and a patient’s potential risk factors while not stereotyping a patient based on those same risk factors.

“[It] made me realize that there needs to be a balance between not letting race and ethnicity bias your opinions and also taking race and ethnicity into account to provide the best care for your patients.”

A few students (6%) noted feeling that passive reinforcement of implicit bias in medical education was commonplace. The common example given was that race was mentioned only when the patient was not White, implying that unless otherwise stated that the default and thus the norm was White.

Themes related to *how the medical profession might address the issue of implicit bias* began with more than four-fifths (84%) of the students who reflected on the IAT expressing the feeling that bias needs to be acknowledged and three-quarters (75%) of them noted that this can be done through making conscious efforts to recognize it. Nearly one-fifth (19%) proceeded to write that they believe it is the physician’s responsibility to advocate for dismantling institutional bias.

“I believe that as physicians we have a responsibility towards the public good and the health of the population and I think that part of this purview is working to undo the injustices that have become established in our society.”

Almost one-quarter (22%) wrote that they felt a call to act on the problems causing disparities and noted the importance of contributing to the work needed both in healthcare and society at large to combat institutional bias.

“The question evolves from how can I be less racist, to how can I help our institutions be less racist?”

A very small proportion (4%) commented that while they thought discussion of implicit and institutional bias was valuable, there needed to be more concrete evidence and quantitative research to truly move forward with combating racism in medicine. One student concluded that more work remains to be done to engage students and clinicians.

“I think that education and discussion around issues like this is an incredibly important thing in our training, but it can also be a difficult thing to get earnest participation in.”

As another student, apparently frustrated by his/her own powerlessness to engage some of his/her reluctant colleagues in this topic put it:

“We dutifully learn about rare cancers which we will likely never see and genetic disorders only described in a handful of patients, which we will certainly never see and yet race, which we will see every day for the rest of our careers is a topic that only manages to draw a small group of students. I can't help but feel that the apathy towards the subject of race … is a part of what has wrought the shocking disparities we are faced with today in healthcare.”

## Discussion

This study achieved its objective of having students include reflections on 1) implicit bias having the potential to influence decisions within their newly chosen profession and 2) where they thought responsibility lay for reducing implicit bias’s effects. This objective was achieved through analyzing the content of two cohorts of students’ required reflective essays resulting from three hours of bias and stereotyping instruction and discussion.

The majority (74%) chose to reflect on the IAT and bias. In addition, they seem to have reflected frankly on their perceptions of implicit biases and the role they can play in clinical decision-making. The students may have been open to this as, in accordance with at least one study’s recommendation, this reflective essay was a personal exercise and did not include a foundational anchor related to normative expectations of physicians [19]. The fact that the exercise was part of a required course and not an elective may have contributed to students approaching this work as normative in medical education. As a required exercise with several options for focusing one’s reflection, this work demonstrates that early in their education, medical students are open to accepting and considering the implications of bias in medicine and within themselves.

Overall, students discussed their experience with the IAT and how that experience created an opportunity for self-reflection and newfound appreciation of their own internalized biases. They also expressed a desire for further training on this subject as well as a need for action and advocacy from themselves as clinicians but also from their colleagues to achieve changes to institutionalized biases and improve future care for patients. There is no empirical evidence in any sector that shows use of the IAT reduces personal bias long term; self-reflection, however, may help individuals become aware of their own biases and take steps to reduce the impact of bias on behavior. Steps to mitigate the impact of bias on behavior may include reducing discretion in decision-making, collecting data to identify inequities, and using checklists. [25]

Physicians have always served as advocates for their patients; indeed, this advocacy role has been institutionalized as a competency to achieve at the University of Massachusetts Medical School [26] and has been mandated by the Royal College of Physicians and Surgeons of Canada [27]. The role of the physician in advocating for broader institutional and cultural change, however, has been the subject of heated debate [28, 29]. Students in this study cohort were motivated to comment on the role of physician advocacy related to countering institutional and personal bias, which creates a sense that momentum towards physicians playing a key role in institutional change exists and could be building. Whether and how students may assume this type of advocacy role is an empirical question that should be studied. Given society’s heightened awareness of the role negative implicit bias plays in a wide range of settings, there is an urgency to determining answers to these questions.

Some students identified the pre-clinical years as an ideal time for addressing issues of implicit bias; nevertheless, few explicitly outlined how they would like to see medical education address implicit and institutionalized bias. While identifying additional curricular time is often seen as an insurmountable barrier, the issue of implicit bias could be threaded throughout existing curricula. One method to integrate implicit bias into existing curricula would be through modifying elements of the patient vignettes frequently used for educational purposes in order to remedy what may be passive reinforcement of implicit biases, particularly to introduce counter-stereotypical information that does not reinforce racial/ethnic stereotypes and inclusion of non-White patient race and ethnicity when it does not affect the patient’s diagnosis or treatment plan [30,31]. An additional approach could be to review all curricula for bias and stereotyping in materials and assessment of what had been left omitted [32]. This could be accomplished through faculty self-reviewing materials or through a school’s curriculum committee, inclusive of student representatives, conducting the review.

Our brief curriculum and assignment stimulated reflection. Preclinical medical students, have not yet been fully exposed to the institutional culture of stereotyping of patients on the wards and in the outpatient clinics that may reinforce bias and, therefore, may lack experiential insight into the practice of medicine and the reality of a typical clinical day’s pressures [33]. Other studies of student perceptions and attitudes have identified preclinical years as an ideal time to intervene, with the acknowledgement that there is a widely documented loss of empathy as students enter their clinical experiences and become assimilated into hospital culture.[34–36] Results from a recent comprehensive study [37] of 3547 medical students at 49 medical schools affirms the importance of early, varied, and continued educational interventions regarding implicit bias. In this study, researchers concluded that medical student experiences within any of the three domains they termed formal curricula, informal curricula, and interracial contact had small but statistically significant associations with changes in reducing implicit racial bias among non- African American students between first and fourth years of medical school. The research found that for students who witnessed attending and resident physicians make negative comments about African American patients, implicit race bias actually increased.

In the past decade, the social determinants of health (SDOH)—the conditions in which people are born, grow, work, live, and age, and the wider set of forces and systems shaping the conditions of daily life [38] have moved from holding a position on the sidelines of health to one closer to center stage. Health care systems and medical educators have accepted the need to address and to influence elements of the SDOH. But as social determinants of health such as housing, education, transportation, and employment are often predicated on the more distal determinant of bias, discrimination, stereotyping, and marginalization, this move upstream must include examining the potential influence of implicit/unconscious bias. Thus, cultural competency curricula have begun to include examining societal and personal bias. Our results indicate that assigning students the IAT with reflection on their experience is a worthwhile curricular element.

This study provides important insight into the attitudes and self-reflections of students’ implicit biases; nevertheless, considerations and limitations are present. The University of Massachusetts Medical School’s Institutional Review Board deemed this body of work “not research.” In educational institutions, it is generally accepted that using either students themselves or their work from a required course to conduct research could be viewed as coercive, therefore sensitivity to potential ethical issues is heightened. Notable issues in the case of the work presented in this article are that 1) the students’ assignment did not require them to write about the IAT (it gave them the option of reflecting on any of the materials that had been part of the class; nevertheless, 74% elected to focus on the IAT in their essays) and 2) the instructors intended the assignment to guide them as they strove to improve one element of the course. Nevertheless, if standards of practice surrounding using either students or their work for research change, it will be critical to ensure ethical standards guide this type of work.

The IAT is a validated tool, yet still faces criticism for its approach to identifying and measuring associations related to bias [39,40]. Authors of the IAT are careful to note that the tool indicates relative association strengths between two concepts but does not reflect actual biases or acts of discrimination related to identified preferences [4]. Moreover, while theme coding accuracy and interrater reliability were high, it remains possible that coders interpreted student responses differently. Students were not explicitly required to reflect on their experience with the IAT or implicit bias; some may have withheld true feelings about the role of implicit bias in medical education due to fear of repercussion to their grades. Additionally, given that these are preclinical students, we cannot measure how these reflections may or may not change future clinical outcomes. Finally, active participation in the curriculum and with the IAT may reflect self-selection bias of students who already exhibit interest in the subject. Despite these limitations, our study found that pre-clinical medical students chose to explore implicit bias through self-reflection. In addition, students expressed motivation to engage in advocacy around bias in medicine.

## Conclusion

Our study demonstrates that medical education curricula can introduce the issue of bias to preclinical medical students who are then able to grapple with and reflect upon issues of implicit bias. Given the extent to which implicit bias permeates our psyches, having medical students early in their education take and reflect on implicit bias through the IAT offers opportunity to confront and address bias. This is likely one necessary—though not sufficient—step among many towards striving for equity in health care. Additional studies of medical students’ perceptions of implicit bias and determinations of how these reflections impact patient l outcomes as students’ progress on their journey to becoming physicians are needed.
